# Deciphering the tight metabolite‐level regulation of glucose‐1‐phosphate adenylyltransferase (GlgC) for glycogen synthesis in cyanobacteria

**DOI:** 10.1111/febs.17348

**Published:** 2024-12-05

**Authors:** Kenric Lee, Sofia Doello, Martin Hagemann, Karl Forchhammer

**Affiliations:** ^1^ Interfaculty Institute of Microbiology and Infection Medicine University of Tübingen Germany; ^2^ Department of Plant Physiology University of Rostock Germany

**Keywords:** 3‐PGA/Pi, biochemical assay, cyanobacteria, GlgC regulation, glycogen synthesis

## Abstract

The enzyme glucose‐1‐phosphate adenylyltransferase (GlgC, EC:2.7.7.27) catalyses the first step in glycogen synthesis by converting glucose‐1‐phosphate into ADP‐glucose, which is added in turn to a growing glycogen chain by glycogen synthases. Thus far, *in vitro* studies of GlgC were mainly performed using colorimetric or radiolabel‐based phosphate release assays, limiting the option for analysing this reaction. With this work, we present a novel *in vitro* continuous assay coupling the subsequent glycogen synthase reaction to the GlgC reaction, thus simulating the process of glycogen synthesis *in vivo*. Using this assay, we revisited GlgC catalytic parameters and screened for metabolites that affect GlgC activity in *Synechocystis* sp. PCC 6803. We also describe in further detail the antagonistic interplay between the GlgC activator, 3‐PGA and the inhibitor, inorganic phosphate, revealing the intricate mechanism by which glycogen formation responds to fluctuations in carbon and energy supply in cyanobacteria.

## Graphical abstract

ADP‐glucose pyrophosphorylase (GlgC) catalyses the rate‐limiting step in glycogen synthesis, converting glucose‐1‐phosphate to ADP‐glucose. Here, GlgC activity was coupled to glycogen synthase (GlgA), whereby ADP‐release causes NADH oxidation through pyruvate kinase (PK) and lactate dehyrdogenase (LDH) reactions, recorded at 340 nm. Reassessment of GlgC catalytic properties revealed a complex interplay between activator molecules (3‐PGA) and inhibitors (inorganic phosphate), showing how glycogen formation responds to carbon and energy fluctuations in cyanobacteria.

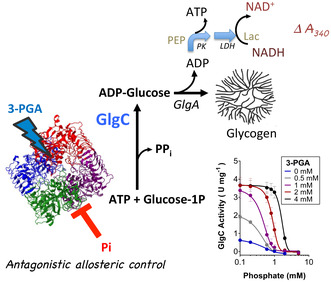

**Abbreviations**
2‐PG: 2‐phosphoglycolate
2‐PGA: 2‐phosphoglycerate
3‐PGA: 3‐phosphoglycerate
ADP‐Glc: ADP‐Glucose
CBB Cycle: Calvin‐Benson‐Bassham cycle
Fru‐1,6P: fructose‐1,6‐bisphosphate
Fru‐6P: fructose‐6‐phosphate
Glc‐1P: glucose‐1‐phosphate
Glc‐6P: glucose‐6‐phosphate
GlgA1: glycogen Synthase 1
GlgC: glucose‐1‐phosphate adenylyltransferase
LDH: lactate dehydrogenase
OPP Cycle: oxidative pentose phosphate cycle
PEP: phosphoenolpyruvate
PGAM: phosphoglycerate mutase
Pi: inorganic orthophosphate
PK: pyruvate kinase
PPi: pyrophosphate
PPiase: pyrophosphatase

## Introduction

Cyanobacteria are a large group of photosynthetic prokaryotes present in nearly every biome on the planet. Given their ability to fix atmospheric CO_2_ via photosynthesis, glycogen is an important storage molecule for carbon assimilation and plays a distinct role in the ability to respond to changing environmental conditions. In cases where nitrogen sources are exhausted, the cells can enter a dormant state known as chlorosis, where they accumulate glycogen, which is later rapidly degraded, once nitrogen is available for a rapid recovery to normal growth [1]. Glycogen degradation serves to provide metabolites for the oxidative pentose phosphate (OPP) pathway, generating reducing equivalents for respiration in the dark, as well as to replenish Calvin‐Benson‐Bassham (CBB) cycle intermediates and other glycolytic shunts once photosynthetic activity is unbalanced or resumes [2, 3]. Furthermore, glycogen degradation can also serve to refill the pool of ADP‐glucose, which is required for the synthesis of the osmoprotectant glucosylglycerol, allowing for better adaptability in changing osmotic conditions [4].

In all cyanobacteria, including the model strain *Synechocystis* sp. PCC 6803 (hereafter *Synechocystis*), the Glucose‐1‐phosphate adenylyltransferase (GlgC, product of *slr1176*, EC:2.7.7.27) catalyses the first committed step in glycogen synthesis, which is the reversible conversion of glucose‐1‐phosphate (Glc‐1P) and ATP to pyrophosphate (PPi) and ADP‐glucose (ADP‐Glc), the immediate precursor for glycogen synthesis. This reaction is rendered irreversible by the action of cellular pyrophosphatases (PPiase), which split the product PPi into two molecules of orthophosphate (Pi). The ADP‐Glc produced is added via a 1,4‐alpha bond to a growing glycogen molecule by glycogen synthase, present as two isoenzymes in *Synechocystis* (GlgA1/*sll0945*, EC:2.4.1.21 and GlgA2/*sll1393*, EC:2.4.1.21). Finally, the branching enzyme (GlgB/*sll0158*, EC:2.4.1.18) introduces 1,6‐branches into the maturating glycogen molecule, increasing the number of potential extension sites for glycogen synthases.

The regulatory and structural properties of cyanobacterial GlgC enzymes have been the subject of extensive study, given their structural and regulatory similarities to those found in plants involved in starch synthesis. The enzyme from cyanobacteria is functionally related to the small subunits of GlgC from photosynthetic eukaryotes, and share a similar sensitivity to changing levels of 3‐phosphoglycerate (3‐PGA) and Pi, which act as an activator and inhibitor, respectively [5, 6, 7, 8]. With that in mind, and given the availability of genetic tools for *Synechocystis*, many efforts were made to improve the productivity of commercially valuable compounds by exploiting and modifying the carbon assimilation pathways present in *Synechocystis* [9, 10, 11, 12, 13].

Despite the key role of GlgC in glycogen synthesis, the current methods employed to study GlgC in the direction of ADP‐Glc synthesis have been limited to discontinuous phosphate release assays using colorimetric methods such as the malachite green assay or radioactive isotope‐based methods, or have other previously reported limitations in terms of sample handling, equipment requirements and cost factor [7, 14, 15, 16, 17, 18]. In this work, we present a new approach to study the GlgC reaction in the direction of ADP‐Glc synthesis. The method combines a continuous spectrophotometric method previously established for GlgA1 with the GlgC reaction [19]. Our method allows for the processing of multiple sets of samples, allowing for the testing of multiple experimental conditions in a single run. Furthermore, the required equipment and reagents for the assay should already be available in most standard research laboratories. As a demonstration, we utilised our assay to further investigate the regulation of GlgC by its allosteric regulators, providing deeper insight into the fine‐tuned regulation of the key reaction in carbon metabolism. We propose that our method could be a useful and easily accessible tool for the study of glycogen synthesis enzymes, and for the modelling of glycogen metabolism *in vitro*.

## Results

### GlgC (Slr1176) gene cloning and sequence analysis

**Fig. 1:**
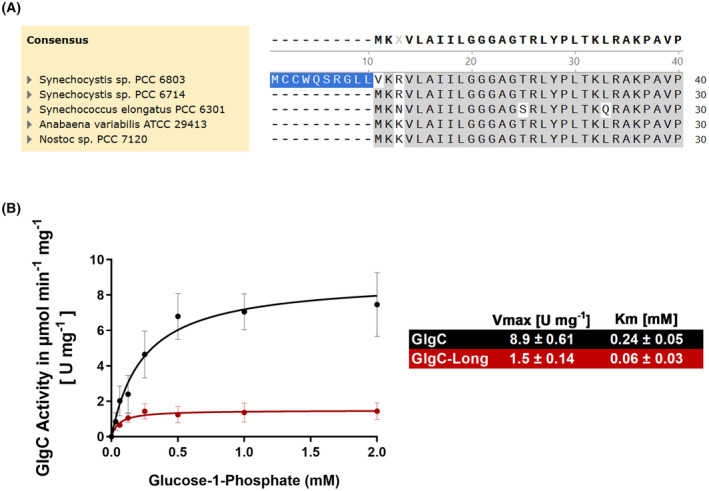
Reannotation of GlgC in *Synechocystis* sp. PCC 6803 with a shorter protein exhibiting higher activity. (A) ClustalW multiple sequence alignment of cyanobacterial GlgC with the first 40 amino acids shown and 10 amino acid N‐terminal extension highlighted in blue. (B) Michaelis–Menten kinetics of GlgC (black) and GlgC‐Long (red). A malachite green assay for free phosphate was used to assess the activity of both variants with Glc‐1P concentrations reflected on the *x*‐axis. The reaction was conducted in the presence of 2 mm 3‐PGA. Each data point represents the mean of at least 3 experiments (*n* = 3) with error bars representing the standard deviation (SD).

The sequence coding for the *Synechocystis* GlgC was retrieved from Uniprot database (P52414). However, using this amino acid sequence in multiple alignment analyses with GlgC proteins from several other cyanobacteria revealed an extension of 10 amino acids at the N‐terminus with a second downstream start codon congruent with those of the other reference organisms (Fig. 1A). The additional 10 amino acids could thus be a result of mis‐annotation, similar to what we experienced with other enzymes from *Synechocystis* [20, 21]. With that in mind, we prepared another pET28a expression construct without the extension and purified both proteins, naming them GlgC and GlgC‐Long, according to their lengths.

### GlgC activity measurements

Figure 1B shows the activity of both GlgC variants in a malachite green assay, where the release of pyrophosphate and its subsequent conversion to orthophosphate from Glc‐1P is colorimetrically quantified after stopping the reaction. GlgC was observed to be more active than GlgC‐long, with specific activities (*V*_max_) of 8.9 ± 0.61 U·mg^−1^ and 1.5 ± 0.14 U·mg^−1^ respectively, when assayed with the substrate Glc‐1P in the presence of the activator 3‐PGA. It is clearly seen that the shorter variant of GlgC is almost 10‐times more active, reinforcing the biological relevance of the shorter variant. Based on this observation we adopted the use of the shorter variant as our standard GlgC.

### Coupling GlgC to the GlgA1 reaction

**Fig. 2:**
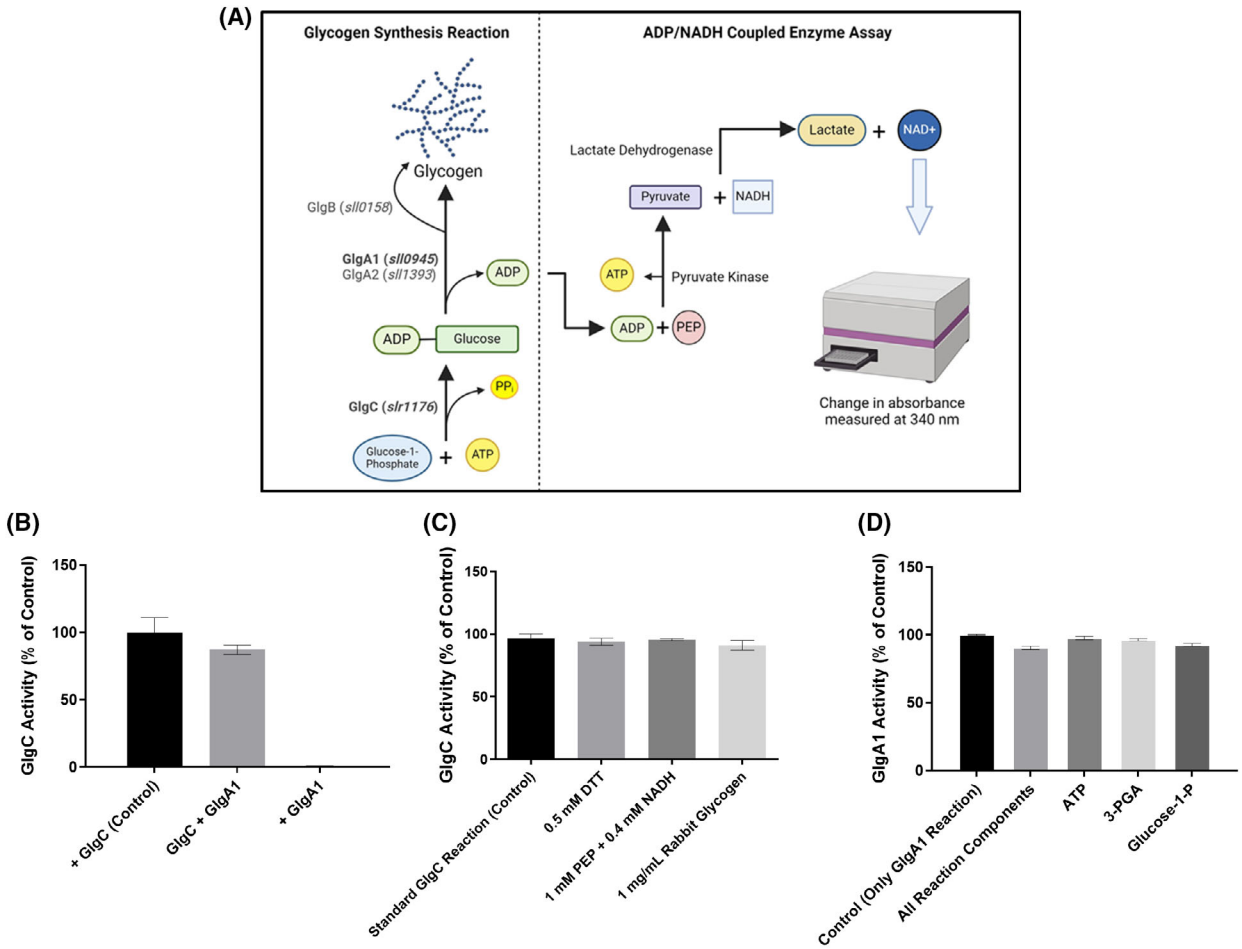
Assay development and Compatibility Testing. (A) Schematic diagram of the glycogen coupled enzyme assay showing the reactions of both GlgC and GlgA1, as well as those corresponding to pyruvate kinase and lactate dehydrogenase of the NADH coupled enzyme system. The cross compatibility of each component was performed using a malachite green assay to assess the impacts of GlgA1 (B) and the components of the GlgA1 coupling assay on the GlgC reaction (C). (D) GlgA1 assay using the ADP/NADH coupling system performed with 2 mm of each tested component of the GlgC reaction, where the reaction was started with 0.25 mm ADP‐Glc. Each data point represents the mean of at least 3 replicate experiments (*n* = 3), with error bars representing the standard deviation (SD).

Assaying the GlgC reaction by the malachite green method has several limitations: the method does not allow real‐time detection of the reaction but requires end‐point measurements, making kinetic analyses less precise than assays with continuous read‐outs. Furthermore, this method prevents the use of phosphate‐containing buffers. To overcome these limitations, we attempted to establish a coupled assay, where the GlgC reaction is subsequently coupled to glycogen synthesis. The concomitant release of ADP from glycogen synthesis can be detected by a standard coupled assay with photometric read‐out (ADP/NADH coupling). Figure 2A presents the design of our entire coupled assay, where Glc‐1P was used as the substrate for the GlgC reaction producing ADP‐Glc, which was added to a growing glycogen chain via the GlgA1 reaction, releasing ADP. The released ADP was quantified, as described previously by Wayllace *et al*. [19], by coupling to pyruvate kinase and lactate dehydrogenase. The entire reaction was spectrophotometrically tracked for absorbance at 340 nm, from which the change in A340 is directly proportional to the amount of released ADP (Fig. 2A). As a first step, we assessed the compatibility of the substrates necessary for the downstream coupled enzyme assay with the GlgC reaction. The GlgA1 protein was also assessed for possible compatibility issues, for all of which no significant interference with the GlgC reaction was observed (Fig. 2B,C). Conversely, the components of the GlgC reaction had also no significant impact on the GlgA1 reaction when assayed alone using the ADP/NADH coupled assay, with ADP‐Glc as the starting substrate (Fig. 2D).

**Fig. 3:**
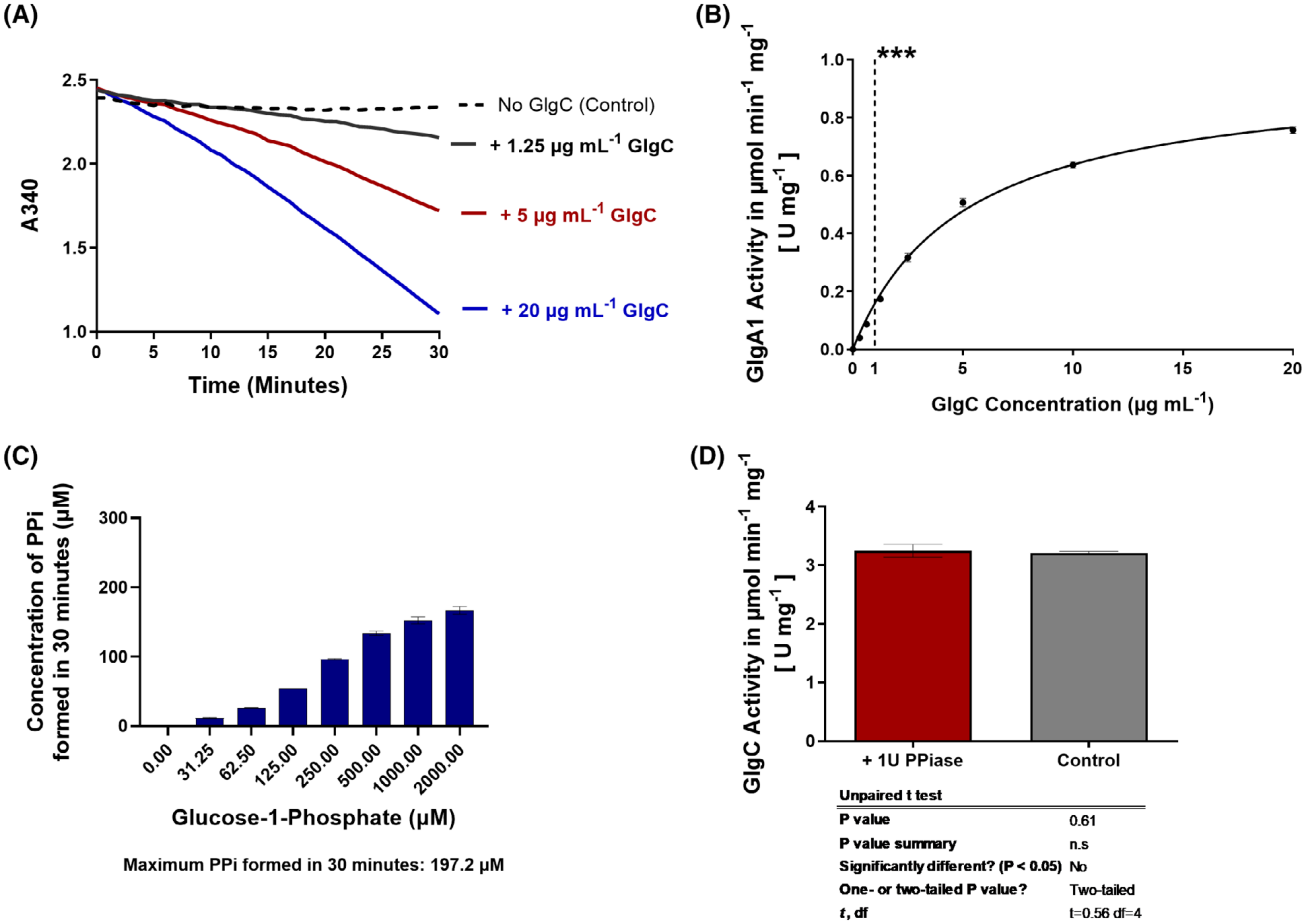
Optimisation of the assay through testing GlgC‐GlgA1 Coupling parameters. (A) Raw reaction curves featuring 3 concentrations of GlgC. GlgC was titrated with 10 μg·mL^−1^ GlgA1 protein. The assay was started with the addition of 2 mm Glc‐1P. (B) Curve showing the relationship between GlgC concentration and GlgA1 activity for the aforementioned assay in part A. (C) Calculated concentration of PPi after 30 min. The assay was started in the presence of 2 mm 3‐PGA by the addition of Glc‐1P at the concentrations on the *x*‐axis. (D) Test for possible effects of 1 U PPiase on the GlgC reaction, controls are samples without PPiase. Where applicable, each data point represents the mean of at least 3 replicate experiments (*n* = 3) with error bars showing the SD. ***1 μg·mL^−1^ GlgC and 10 μg·mL^−1^ GlgA1 were taken as standard concentrations for subsequent assays performed in this work.

After excluding possible incompatibility issues, we analysed the raw reaction curve over 30 min, where NADH oxidation can be spectrophotometrically followed by a decreasing absorption at 340 nm (A340). As shown in Fig. 3A, the change in absorbance was not immediately linear upon starting the reaction by the addition of Glc‐1P but the reaction velocity gradually increased and reached a stable slope after about 15 min. This result shows that the concentration of the GlgC reaction product ADP‐glucose initially increases until an equilibrium between synthesis and consumption is achieved. Next, we needed to identify the appropriate concentration of GlgC in this coupled system to reach a nearly linear relation between GlgC activity and measured velocity of the coupled GlgA1 reaction. Therefore, different amounts of GlgC were titrated in a series of assays containing a fixed concentration of 10 μg·mL^−1^ GlgA1 and starting the reaction with 2 mm Glc‐1P. Fig. 3B shows that the reaction read‐out, in terms of GlgA1 activity, is nearly linear to the amount of added GlgC at low concentration ranges, whereas above 2 μg·mL^−1^ GlgC, the reaction velocity begins to approach saturation. With these considerations in mind, we decided to use 1 μg·mL^−1^ GlgC for further kinetic assays coupled with 10 μg·mL^−1^ GlgA1, which allows sensitive detection of any changes in GlgC activity imposed by substrate and effector molecules. We also investigated the concentration of PPi after a standard 30‐min reaction and found that the highest concentration lies within the micromolar range (Fig. 3C), which is below the concentrations that turned out to be inhibitory (see below). Compared to the malachite green assay, we also excluded PPiase from the coupled assay after verifying that the exclusion of PPiase from the coupled assay setup did not have any significant effect (Fig. 3D).

### Coupled assay kinetic parameters for GlgC are comparable to those of malachite green assays

**Fig. 4:**
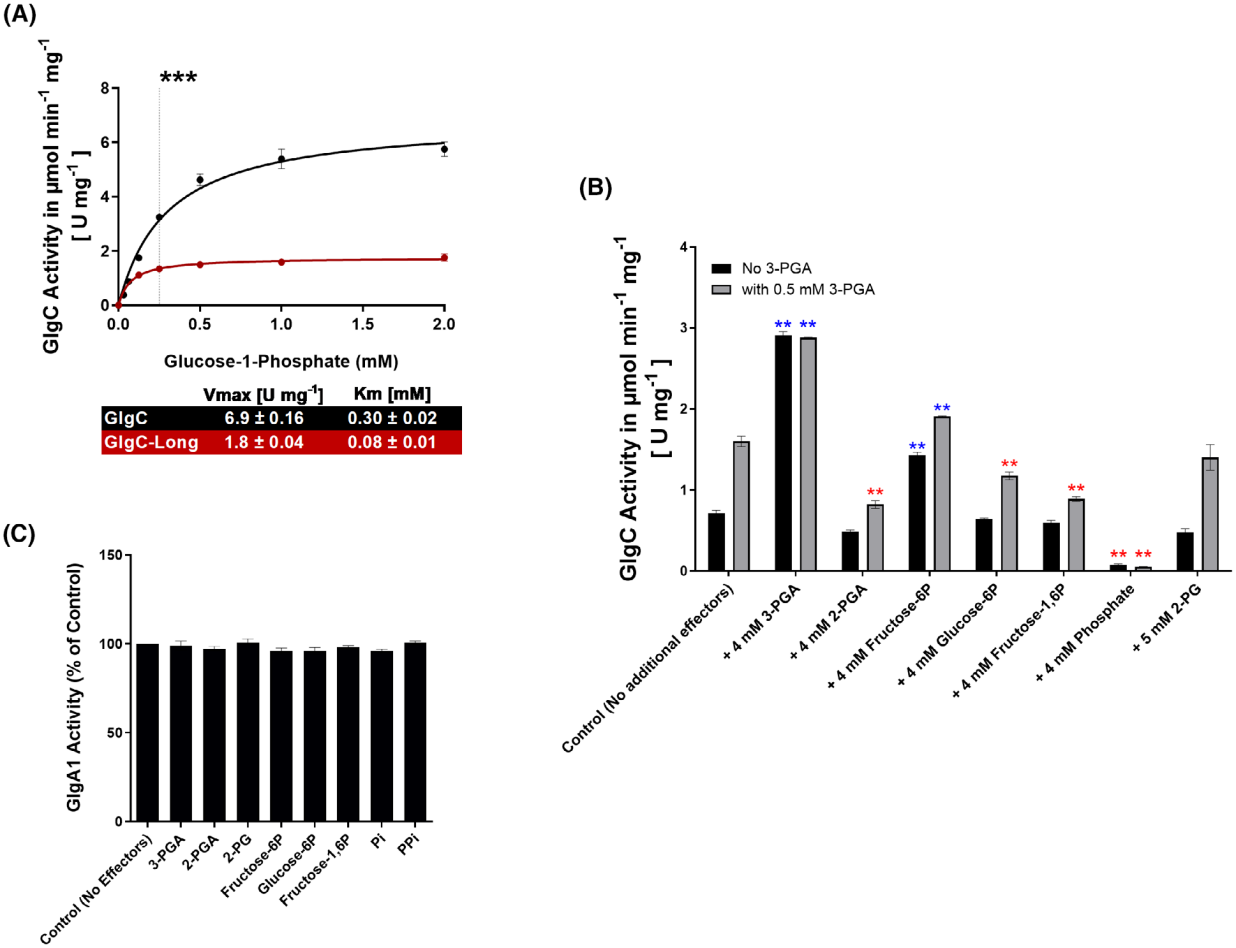
GlgC coupled assay kinetics and effector screening. (A) Michaelis–Menten kinetics of GlgC (black) and GlgC‐Long (red) performed with the coupled assay using the previously determined parameters. The assay was started in the presence of 2 mm 3‐PGA by the addition of Glc‐1P. ***0.25 mm Glc‐1P used for subsequent assays. (B) Screening of potential activators of GlgC, in black are GlgC reactions without activator (3‐PGA), reactions with 0.5 mm 3‐PGA are shown in grey. Additional effectors were added to both groups to screen for potential regulatory effects. **Blue stars represent significant activation vs controls, while red stars represent inhibition. (C) A similar experiment performed using the ADP/NADH coupled assay using ADP‐Glc and GlgA1 to screen for impacts on GlgA1 activity. Where applicable, each data point represents the mean of at least 3 replicate experiments (*n* = 3) with error bars showing the SD.

With the basic parameters of the assay in hand, we compared GlgC with GlgC‐Long in a similar fashion as presented above (Fig. 1C) but using our newly established assay. Figure 4A shows the Michaelis–Menten kinetics of both enzymes. Again, the shorter GlgC protein was observed to be more active than GlgC‐Long. We found that the V_max_ of GlgC was around 20% lower in the coupled assay as compared to the malachite green assay (6.9 ± 0.16 mm compared to 8.9 ± 0.61 mm), whereas the V_max_ of GlgC‐Long was comparable across assays (1.8 ± 0.04 mm compared to 1.5 ± 0.14 mm). The respective Michaelis constant (K_m_) value for the substrate Glc‐1P was slightly lower in the coupled assay as compared to the malachite green assay, for both GlgC and GlgC‐Long variants (GlgC: 0.30 ± 0.02 mm compared to 0.24 ± 0.05 mm, and GlgC‐Long: 0.08 ± 0.01 mm compared to 0.06 ± 0.03 mm).

### Screening for GlgC effectors

**Fig. 5:**
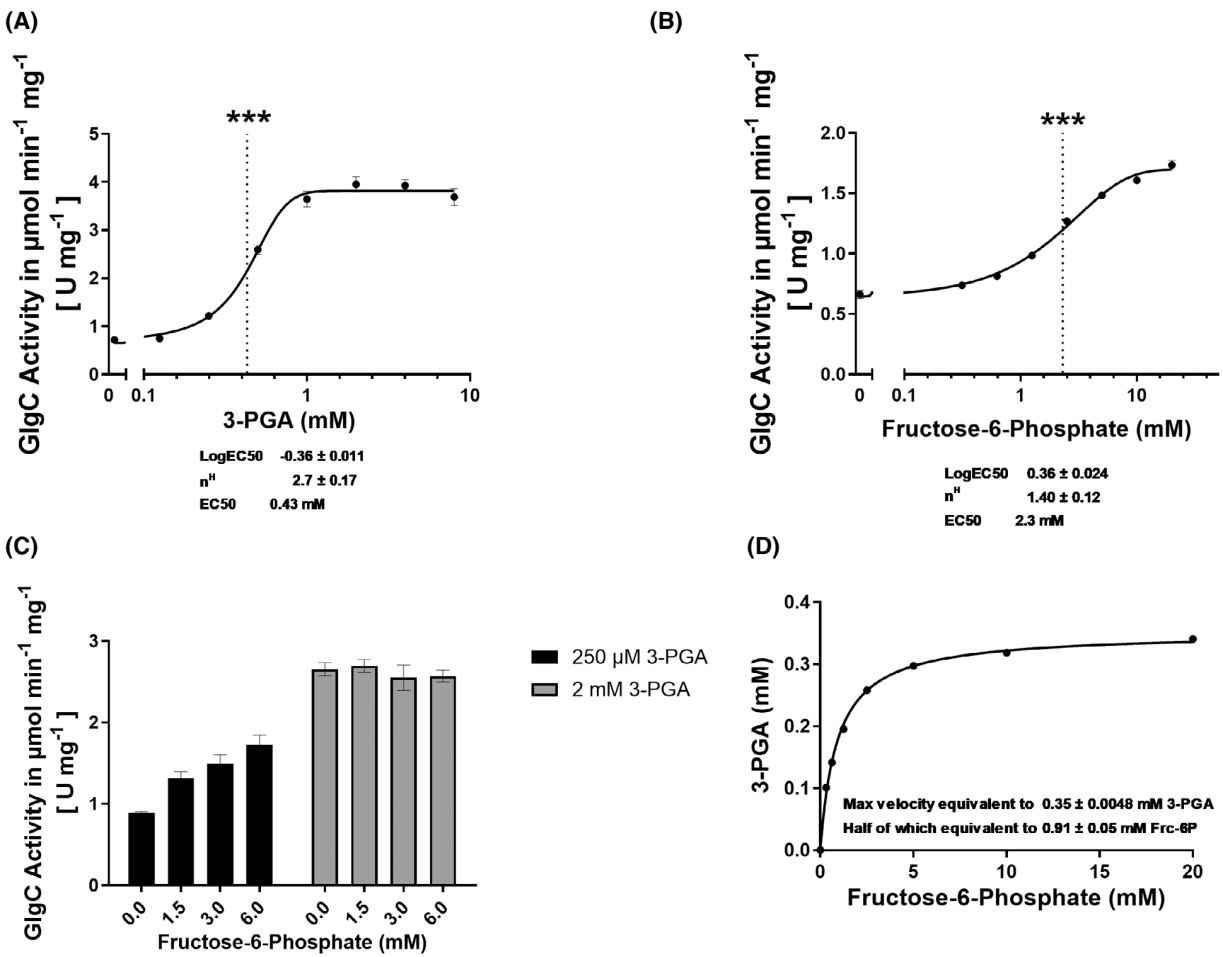
Effect of the GlgC activators: 3‐PGA and Fru‐6P. EC50 curves established using the coupled enzyme assay with varying concentrations of (A) 3‐PGA and (B) Fru‐6P. ***EC50 values of 3‐PGA (0.43 mm) and Fru‐6P (2.30 mm). (C) GlgC activity with increasing concentrations of Fru‐6P in the presence of low and high concentrations of 3‐PGA. (D) Curve showing the equivalent concentration of 3‐PGA corresponding to the concentration of Fru‐6P with regards to GlgC activation. Where applicable, each data point represents the mean of at least 3 replicate experiments (*n* = 3) with error bars showing the SD.

As a first step, we screened for further effector molecules of GlgC activity, by shortlisting a few metabolites that play roles in glycolysis and carbon metabolism in *Synechocystis* (Fig. 4B). To exclude any impacts of these compounds on GlgA1, we also tested the effect of these metabolites, in addition to PPi, on the GlgA1‐based coupling reaction (Fig. 4C), confirming that these molecules do not affect the GlgA1 reaction. Accounting for potential additive effects with the activator 3‐PGA, we also tested these metabolites in the absence of 3‐PGA as well as at a concentration near the calculated EC50 value of 0.5 mm (Fig. 5A). Figure 4B shows that in the presence or absence of 3‐PGA, Pi and 2‐phosphoglycerate (2‐PGA) were observed to be inhibitors whereas fructose‐6‐phosphate (Fru‐6P) activated the reaction. Moreover, glucose‐6‐phosphate (Glc‐6P) and fructose‐1,6‐biphosphate (Fru‐1,6P) were observed to also act as mild inhibitors of GlgC in the presence of 3‐PGA, in addition to those already mentioned above. The metabolic indicator of carbon limitation, 2‐phosphoglycolate (2‐PG), was found to have no effect on the GlgC reaction.

To further quantify the activating effect of 3‐PGA, we titrated 3‐PGA in the GlgC reaction and started the assay with the previously defined concentration of Glc‐1P close to K_m_ (0.25 mm). To determine the EC50 for 3‐PGA, the measured GlgC activity was fitted to logarithmic 3‐PGA concentrations with variable slopes, yielding an EC50 of 0.43 mm and an eight‐fold activation of the GlgC reaction at saturating activator concentrations was determined (Fig. 5A).

Next, to more closely analyse the activating effect of Fru‐6P, we first assessed the activation of the GlgC reaction in the absence of 3‐PGA. Fitting the data as described above for 3‐PGA, revealed an EC50 of 2.3 mm and an approximate three‐fold increase in activity at saturating concentrations of Fru‐6P (Fig. 5B). In the presence of low levels of 3‐PGA, Fru‐6P was able to further activate GlgC, but when 3‐PGA was present at saturating concentrations, no further activation of GlgC reaction occurred (Fig. 5C). The equivalent concentrations of 3‐PGA corresponding to the same degree of activation of GlgC as caused by Fru‐6P were estimated by comparing the activation curves of 3‐PGA and Fru‐6P and fitting the corresponding values as shown in Fig. 5D. It was observed that excess Fru‐6P can only achieve as much activation as 0.35 mm 3‐PGA, which is slightly under the calculated EC50 value of 3‐PGA of 0.43 mm.

### 2‐PGA acts as a negative regulator of GlgC

**Fig. 6:**
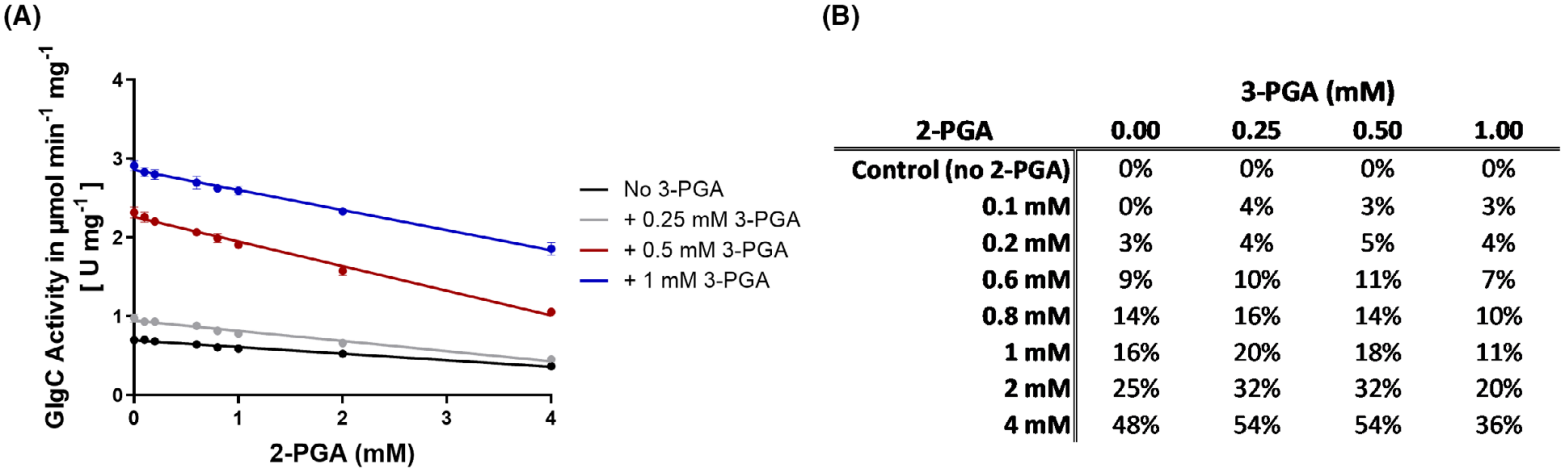
2‐PGA inhibition of GlgC. (A) 2‐PGA inhibition curves of GlgC in the presence of the listed concentrations of 3‐PGA. (B) Accompanying table showing the percentage inhibition of GlgC activity. Samples not treated with 2‐PGA were taken as controls. Where applicable, each data point represents the mean of at least 3 replicate experiments (*n* = 3) with error bars showing the SD.

Contrary to previously reported findings [7], our effector molecule screening showed a negative effect of 2‐PGA on GlgC activity (Fig. 6A). 2‐PGA substantially inhibited GlgC at higher concentrations. However, when compared to Pi, 2‐PGA inhibition appeared significantly weaker, with no complete inhibition of GlgC achievable with the concentration range tested (Fig. 6B). Furthermore, in contrast to the inhibitor Pi, inhibition by 2‐PGA did not exhibit any cooperativity with 3‐PGA, given by the linear decrease in GlgC activity with increasing 2‐PGA concentrations seen in Fig. 6.

### 3‐PGA lifts pi inhibition

**Fig. 7:**
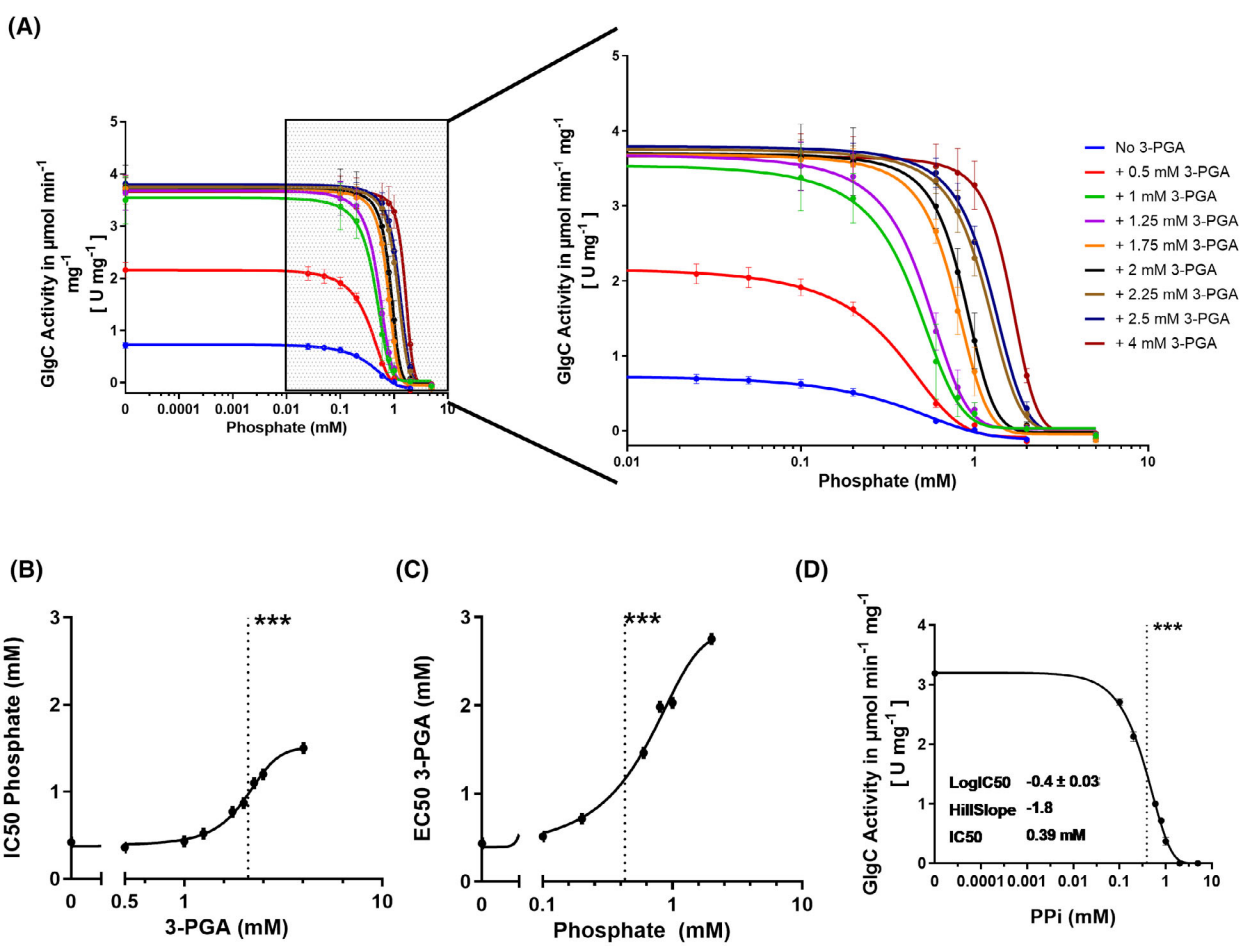
Modulation of GlgC Inhibition by 3‐PGA. (A) Phosphate inhibition curves of GlgC with increasing concentrations of 3‐PGA. The coupled assay was used to evaluate the effect of increasing concentrations of phosphate on the GlgC reaction in the presence of increasing 3‐PGA concentrations. (B) Pi IC50 in relation to increasing 3‐PGA concentration. ***At 2.1 mm 3‐PGA, the IC50 of phosphate is increased by half. (C) 3‐PGA EC50 in relation to increasing Pi concentration. ***At 0.43 mm Pi, the EC50 of phosphate is increased by half. (D) PPi inhibition curves of GlgC in the presence of 2 mm 3‐PGA. ***IC50 of PPi is 0.39 mm at 2 mm 3‐PGA. Where applicable, each data point represents the mean of at least 3 replicate experiments (*n* = 3) with error bars showing the SD.

The role of Pi in GlgC regulation has previously been described using discontinuous assay methods [5, 7]. With our novel continuous assay in hand, we revisited the inhibitory effects of Pi in greater detail. Figure 7 shows the interplay between the key activator 3‐PGA and the inhibitor Pi via a series of Pi inhibition curves at increasing 3‐PGA concentrations. Immediately apparent is that Pi concentrations in the millimolar range led to an almost complete inhibition of GlgC. Furthermore, we observed that the inhibition of GlgC by Pi is strongly modulated by 3‐PGA concentrations in a non‐linear manner. Plotting the IC50 of phosphate inhibition against the respective 3‐PGA concentrations present in the assay revealed a sigmoidal relationship between 3‐PGA concentration and the IC50 of Pi, indicating cooperative behaviour in the antagonistic relationship between Pi inhibition and 3‐PGA activation of GlgC (Fig. 7B). Conversely, plotting the EC50 for 3‐PGA in the presence of varying Pi concentrations also showed a similar sigmoidal relationship (Fig. 7C). Table 1 shows that this relationship also results in greater Hill coefficient (n^H^) of the Pi inhibition curves at higher 3‐PGA concentrations, indicating that 3‐PGA directly affects the cooperativity of GlgC towards Pi inhibition. We also further examined the potential inhibitory effect of PPi, revealing that PPi is a potent inhibitor of GlgC. In the presence of 2 mm 3‐PGA, an IC50 of 0.39 mm PPi was determined, compared to 0.87 mm for Pi at the same concentration of 3‐PGA (Fig. 7D). This indicates that PPi is inhibiting approximately twice as effective as Pi and that whether PPi is converted into two molecules of Pi or remains unhydrolysed does not impact the assay. Furthermore, we note that the concentration of PPi generated during a given reaction is still well below the IC50 value (Figs 3C and 7D), indicating that PPi produced in this manner has little to no impact on GlgC regulation.

**Table 1:** Percentage inhibition of GlgC by Pi at the given concentrations in the presence of the stated concentrations of 3‐PGA. IC50 and hill constant (n^H^) values for Pi are also listed at the given concentrations of 3‐PGA. Samples without Pi were taken as controls.

Phosphate (pi)	3‐PGA (mm)									
	0.00	0.50	1.00	1.25	1.75	2.00	2.25	2.50	3.00	4.00
Control (no Pi)	0%	0%	0%	0%	0%	0%	0%	0%	0%	0%
0.025 mm	3%	3%	–	–	–	–	–	–	–	–
0.05 mm	6%	5%	–	–	–	–	–	–	–	–
0.1 mm	11%	11%	4%	3%	3%	2%	1%	2%	2%	1%
0.2 mm	25%	23%	11%	7%	5%	4%	3%	3%	3%	2%
0.6 mm	71%	78%	72%	63%	28%	19%	11%	10%	8%	5%
0.8 mm	–	–	86%	83%	55%	42%	22%	18%	16%	7%
1 mm	85%	91%	92%	91%	76%	66%	38%	33%	29%	11%
2 mm	100%	100%	97%	98%	96%	95%	93%	91%	93%	79%
5 mm	–	–	100%	100%	100%	100%	100%	100%	100%	100%
Phosphate IC50	0.42	0.36	0.43	0.52	0.77	0.87	1.10	1.20	1.20	1.50
n^H^	−1.70	−2.20	−2.90	−3.60	−4.40	−4.30	−4.00	−4.10	−4.70	−5.00

### Binding of effector molecules promotes GlgC tetramerisation

**Fig. 8:**
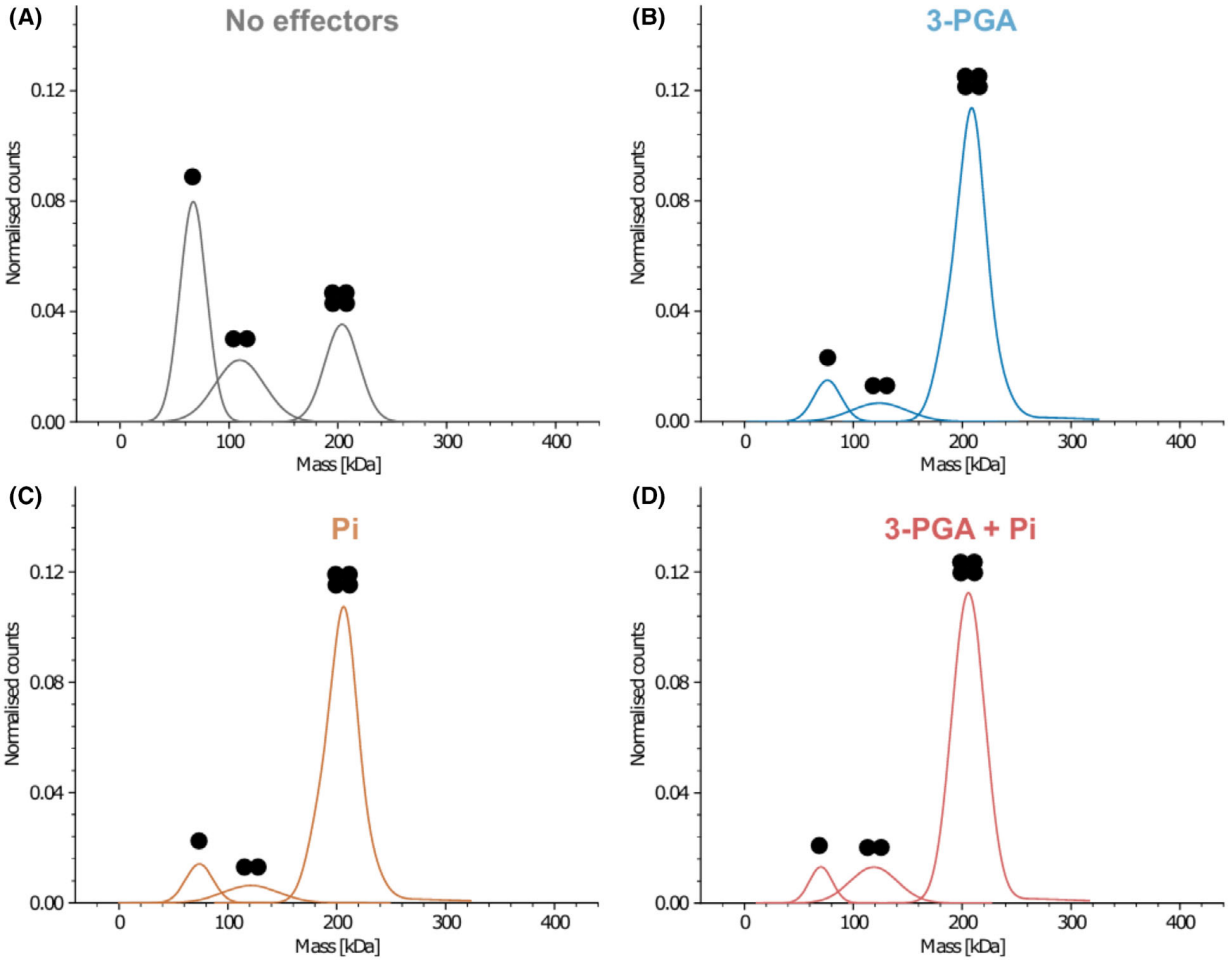
Mass photometry analysis of the oligomeric state of GlgC. GlgC monomer mass 49.3 kDa. Oligomers are denoted by the respective number of black points above each corresponding peak. (A) Oligomerisation states of GlgC in the absence of effectors. (B) With 2 mm 3‐PGA. (C) With 2 mm Pi. (D) With 2 mm 3‐PGA and 2 mm Pi.

Given that *Synechocystis* GlgC is known to be active in its tetrameric form, we analysed the effect of the activator 3‐PGA and inhibitor Pi on its oligomeric state using mass photometry [22]. In the absence of effectors, GlgC was found to be mainly present as monomers, with dimers and tetramers being far less prevalent (Fig. 8A). Upon incubation with the effectors 3‐PGA or Pi, the distribution of the species shifted to favour the assembly of tetramers (Fig. 8B,C). This indicates that both the inhibitor and the activator promote tetramerisation, where the activator promotes the formation of an active tetramer, whereas the inhibitor Pi promotes the assembly of an inactive tetrameric form. In agreement with this observation, the concurrent presence of Pi and 3‐PGA did not further impact oligomerisation, with the tetrameric form of the enzyme being present regardless of which allosteric effector is present (Fig. 8D). Together, it can be assumed that the tetrameric state of GlgC is the physiologically relevant state due to the crowded cellular environment and the constant presence of allosteric effectors stemming from various cellular processes.

## Discussion

We constructed an *in vitro* assay by coupling the GlgC reaction with that of GlgA1 and adapting a previously established ADP/NADH‐coupling system [19]. Using this assay, we studied in detail the synthesis of ADP‐Glc from Glc‐1P by GlgC, the first step in glycogen synthesis. We took advantage of the continuous spectrophotometric design of the assay to further study the interactions between selected regulators of GlgC.

The coupled assay detects the formation of ADP‐glucose by the subsequent release of ADP via GlgA1 reaction. Compared to the malachite green phosphate release assay, this more precise measurement revealed slightly lower *V*_max_ values (Malachite green: 8.9 ± 0.61 U·mg^−1^, Coupled assay: 6.9 ± 0.16 U·mg^−1^), resulting from a significant reduction in the standard deviation of the measurements, and in particular, a more precise assessment at low substrate concentrations, when comparing Figs 1B to 4A. Additionally, the assay overcomes several technical disadvantages of the non‐continuous, multi‐step design of the malachite green assay, where the reaction has to be stopped after selected time points and samples drawn for incubation with the malachite green solution prior to measurement. This presents a technical challenge when studying a reaction over time, where the window between sampling and measurement can be especially small and prone to errors, growing with increasing sample numbers, in addition to disruptions in the assay system itself when samples are drawn. The coupled assay is also a safer and more accessible alternative to radioactive label‐based methods, producing comparable results with the aforementioned advantages [18].

Taking advantage of these improvements, we were able to validate the role and fine interplay between key regulators of GlgC, 3‐PGA and Pi, as well as screen other previously reported metabolites. The possibility of high‐resolution reaction curves without technical interference of Pi when added as an effector molecule allows for the real‐time observations of GlgC inhibition by Pi to be made. Interestingly, we found that PEP had no impact on the GlgC at tested concentrations and that 2‐PGA acted as a weak inhibitor of GlgC while they were both previously reported to be slight activators of GlgC in *Synechocystis* [7].

### GlgC is regulated by 3‐PGA and pi antagonism

The role of 3‐PGA as a cyanobacterial GlgC activator has been firmly established, in addition to the inhibitory role of Pi [5, 7, 23]. However, the significance and dynamics of Pi inhibition was not further addressed for cyanobacteria despite the abundance of relevant work on plant GlgC. Using the newly established assay we were able to study Pi in more detail and expand upon its relation to 3‐PGA activation of *Synechocystis* GlgC.

As an important first step, we demonstrated that the increase in GlgC activity was observed to be up to a maximum of eight‐fold at concentrations of above 1 mm 3‐PGA. This activation also corresponds to the increase in GlgC tetramerisation (Fig. 8A) [22]. Previous studies with *Synechocystis* showed that the cellular amount may vary between 1 and 5 mm when cultivated under continuous light and high or ambient CO_2_ conditions [24]. Experiments with *Synechocystis* under diurnal conditions revealed that 3‐PGA levels were found to be more than two‐fold higher during light phases when the CBB cycle is active compared to levels in the dark [25]. These values lie within the ranges at which we observe full activation of GlgC and EC50 value of 3‐PGA, respectively. Should these findings be further validated, this could firmly establish the link between light/dark regulation of GlgC via 3‐PGA.

**Fig. 9:**
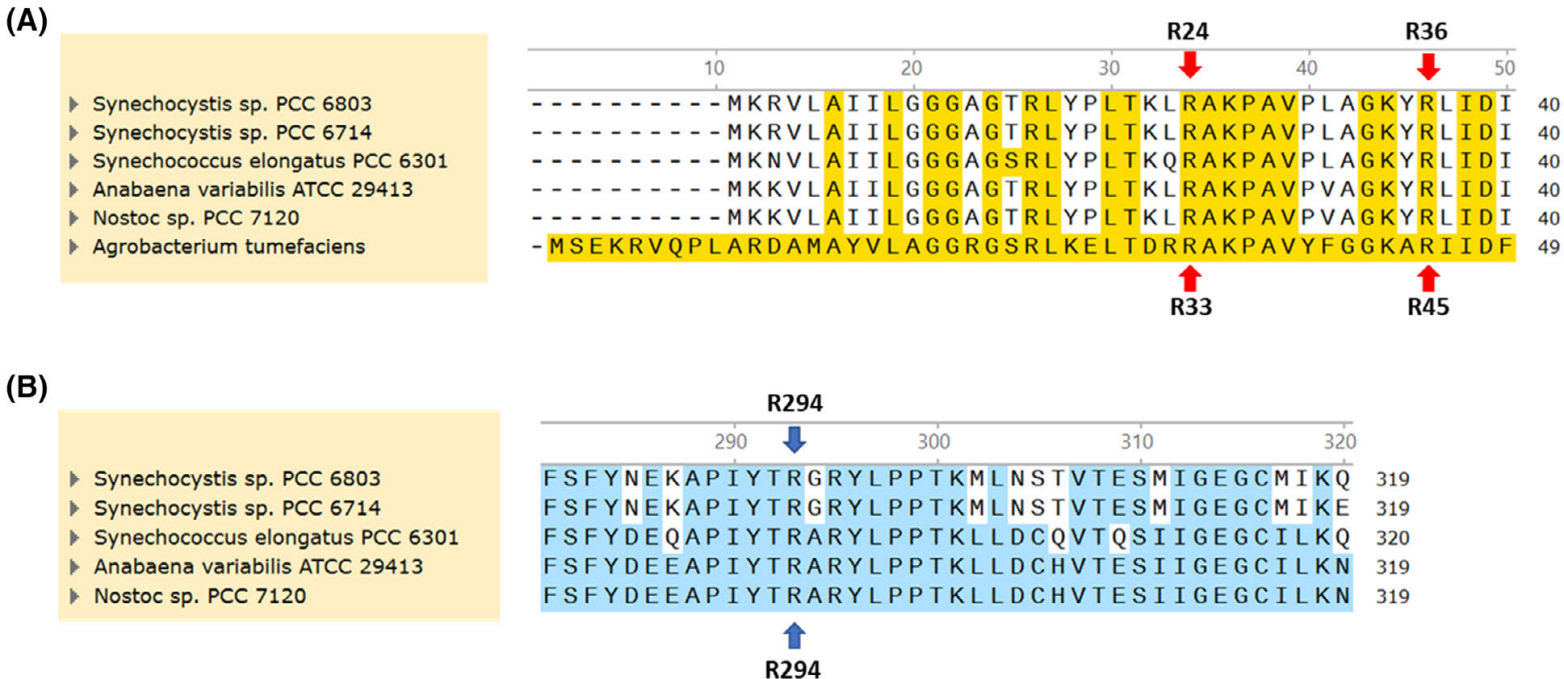
ClustalW multiple sequence alignment of cyanobacterial GlgC. (A) N‐terminal amino acids shown with conserved served sequences highlighted in yellow, compared to *Agrobacterium tumefaciens*. Conserved arginine sites responsible for Fru‐6P and Pi binding, R33 (R24) and R45 (R36) are indicated with red arrows. (B) C‐Terminal amino acids shown with conserved served sequences highlighted in blue. The conserved arginine residue (R294) responsible for inhibitor selectivity is indicated by blue arrows.

With our assay, we revealed a marked antagonism based on cooperative interactions between Pi inhibition and 3‐PGA activation (Fig. 7A,B). The sigmoidal increase in the IC50 of Pi caused by 3‐PGA, along with increasing Hill coefficient of the cooperative phosphate inhibition (Table 1) indicates a mechanism by which 3‐PGA alleviates Pi inhibition by decreasing the affinity of the respective allosteric regulatory sites for Pi. This relationship appears to be reciprocal, such that Pi also increases 3‐PGA EC50 in a similar manner (Fig. 7C). These findings agree with the observations from previous work on GlgC from *Anabaena* (*Nostoc*) sp. strain PCC 7120, indicating that this regulatory mechanism might be shared among cyanobacteria. The antagonism between the effectors Pi and 3‐PGA also suggests that they act at nearby sites that directly influence each other. Two arginine sites in the N‐terminal region (R33 and R45) of GlgC from *Agrobacterium tumefaciens* were previously shown to be the binding sites for the inhibitor Pi [26]. A multiple alignment with GlgC from cyanobacteria showed conservation of the respective residues, corresponding to R24 and R36 in GlgC from *Synechocystis* (Fig. 9A). The binding sites of 3‐PGA have also previously been identified as two lysine residues (K382 and K419) near the C‐terminus of the enzyme by the group of Jack Preiss [8, 27, 28]. Furthermore, these previous studies revealed by site‐directed mutagenesis, a conserved arginine residue near the C terminus (R294) in GlgC from *Anabaena* (*Nostoc*) sp. strain PCC 7120 that plays an important role in inhibitor selectivity and also impacted 3‐PGA binding and activation (Fig. 9B) [29].

**Fig. 10:**
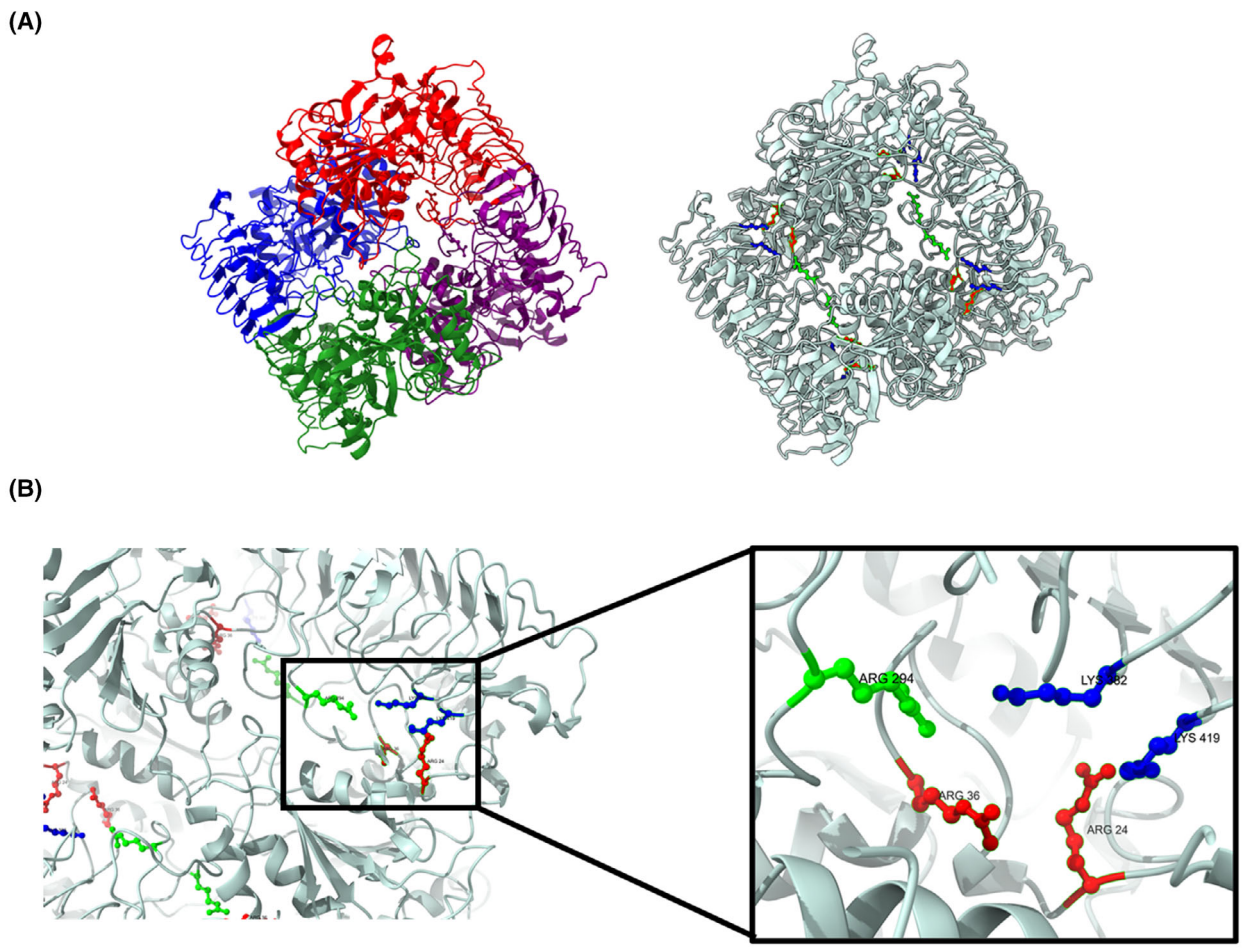
GlgC tetrameric structure predicted using Alphafold2. (A) Structure predictions of GlgC showing the individual GlgC monomers in different colours (left), and the binding sites of the effectors (right). (B) Expanded view showing the side chains of Lys382, Lys418 responsible for 3‐PGA binding (blue), and Arg24, Arg36 for Pi (red) as well as Arg294 responsible for effector selectivity (green).

Modelling these conserved effector binding sites with alphafold2 predicted a tetrameric structure of GlgC which revealed the close proximity of these sites to each other (Fig. 10) [8, 27, 28]. Thus, the model provides straight‐forward mechanistic explanation for the experimental findings when assuming that the antagonism between 3‐PGA activation and Pi inhibition results from the interference between their binding sites. A similar mechanism is present in the GlgC of *Escherichia coli*, where the allosteric sites of its activator Fru‐1,6P and inhibitor AMP were shown to partially overlap, leading to the protection of the opposite binding site when an effector is bound [30]. Given our observation that the presence of Pi did not prevent GlgC tetramerisation, but instead also promoted it, indicates that the inhibitory effect of Pi in GlgC is not a simple reversal or prevention of 3‐PGA facilitated tetramerisation. A similar mechanism to that of the *E. coli* GlgC could be conceivable for Synechocystis GlgC, where the binding of either 3‐PGA or Pi to their respective could antagonise the binding of the other effector without impacting the assembly of the GlgC homotetramer. Given the location of R294 between the allosteric sites of both effectors, it is reasonable to suggest that R294 could play a role in coordinating the binding of effectors or might facilitate the effects of their binding.

Additionally, we also found that the inhibition of GlgC by PPi is twice that of Pi, although it is unlikely that it plays a direct regulatory role due to the action of cellular pyrophosphatases. With that in mind, while the PPi generated during the formation of ADP‐Glc might contribute to GlgC regulation directly or via the release of two molecules of Pi, the concentration of PPi produced in an ongoing reaction is well below inhibitory levels (Figs 3C and 7D). Furthermore, under energy sufficient conditions, the released Pi is immediately reused in other cellular processes such as ATP regeneration, allowing for the uninhibited GlgC reaction to occur. On the contrary, energy limitation or situations that could lead to increased Pi levels would result Pi accumulation arising from ADP‐Glc synthesis and GlgC inhibition could occur.

### 2‐PGA and Fru‐6P contribute to GlgC regulation in response to changing conditions

In *Synechocystis*, nitrogen deprivation, sensed by the PII signalling protein as increased 2‐oxoglutarate levels, results in PirC‐mediated phosphoglycerate mutase (PGAM) inhibition and the subsequent accumulation of 3‐PGA [20]. This accumulation of 3‐PGA drives the carbon flux in the direction of glycogen synthesis. In this situation, elevated 3‐PGA concentrations could be essential to balance and maintain the full activation of GlgC in light of the constant regeneration of the Pi pool from ADP‐Glc synthesis via cellular PPiases, and those arising from other cellular processes. An increase in 3‐PGA levels, caused by an increase in carbon fixation via the CBB cycle, would also result in a shift towards glycogen synthesis by the activation of GlgC.

The product of RubisCO 3‐PGA is converted into 2‐PGA by the PGAM reaction, which funnels newly fixed carbon into lower glycolysis, where it is directed towards anabolic pathways such as amino acid or fatty acid synthesis. 2‐PGA is also generated from the oxygenase reaction of RubisCO, where 2‐PGA is the final product of photorespiration in *Synechocystis* [31]. The high concentrations at which 2‐PGA exerts a noticeable inhibition of GlgC could therefore be relevant under conditions of photorespiration, given that 2‐PGA was found to accumulate in the millimolar concentration range under low carbon conditions [24]. Under these conditions, carbon is lost rather than assimilated and glycogen degradation acts to refill the CBB metabolite pool [3]. The linear relationship observed between GlgC inhibition and 2‐PGA concentration, together with the structural similarities with 3‐PGA, could indicate that 2‐PGA might be binding to the same allosteric sites as 3‐PGA. Regulation by 2‐PGA could be an additional layer of regulation for fine‐tuning glycogen synthesis, with the main inhibitor being Pi.

We also found that Fru‐6P is able to activate GlgC, albeit to a lesser extent than 3‐PGA, which could indicate that Fru‐6P could also occupy the same allosteric sites as 3‐PGA and activate GlgC. Supporting this hypothesis was our observation of an additive effect on GlgC activation by Fru‐6P at lower 3‐PGA concentrations. However, once 3‐PGA is present at saturating concentrations, no further activation by Fru‐6P occurs. This shows that the preferred activator is still 3‐PGA (Fig. 5). An alternative model for this observation could be that Fru‐6P binds to a separate allosteric site that only results in a maximum activation of GlgC which less than that of 3‐PGA. The *A. tumefaciens* GlgC is regulated by Pi and Fru‐6P, instead of 3‐PGA. It has been shown that the previously discussed conserved arginine sites in the N‐terminal region responsible for Pi binding are also responsible for activation by Fru‐6P [26]. This might provide hints that the same regulatory sites could also contribute to the activation of GlgC by Fru‐6P in *Synechocystis*. We propose that under certain nutritional conditions, such as photomixotrophic conditions, Fru‐6P could act as an alternative activator of GlgC when 3‐PGA levels are low adding another layer of regulation for carbon entry into the glycogen cycle. By contrast, under conditions of nitrogen‐limitation, the inhibition of PGAM activity by the PII‐controlled effector protein PirC results in an increase in 3‐PGA, which overrides the effect of other effector molecules, leading to a drastic increase in glycogen synthesis.

## Materials and methods

### Culture and growth conditions

Unless otherwise specified, liquid cultures were grown at 37 °C with shaking at 125 rpm in Luria‐Bertani (Lennox) medium with appropriate antibiotics, where applicable. Plate cultures were grown under similar conditions with the addition of 1.5%_(w/v)_ agar. All *E. coli* strains are listed in Table 2.

**Table 2:** *Escherichia coli* strains used for cloning and protein expression.

Strain: *E. coli* NEB10β
Genotype: *Δ(ara‐leu) 7697 araD139 fhuA ΔlacX74 galK16 galE15 e14‐ φ80dlacZΔM15 recA1 relA1 endA1 nupG rpsL (StrR) rph spoT1 Δ(mrr‐hsdRMS‐mcrBC)*
Strain: *E. coli* Rosetta‐gami (DE3)
Genotype: *Δ(ara‐leu)7697 ΔlacX74 ΔphoA PvuII phoR araD139 ahpC galE galK rpsL (DE3) F′[lac + lacIq pro] gor522::Tn10 trxB pRARE2 (CamR, StrR, TetR)*

### Expression and purification of recombinant his‐tag proteins

Glycogen synthesis genes from *Synechocystis* sp. PCC 6803 were amplified from genomic DNA via PCR using primers 1–4 specific for GlgA1 or GlgC, with overlap extensions for Gibson assembly (Table 3). The pET28a plasmid backbone was similarly linearised and amplified using PCR with primers 5–8. Gibson assembly was used to assemble the final expression constructs for GlgA1 and GlgC, which were used to transform electrocompetent *E. coli* NEB10β cells for selection with 50 μg·mL^−1^ kanamycin. Positive clones were verified via sequencing and transformed into electrocompetent *E. coli* Rosetta‐gami cells for protein expression.

**Table 3:** List of PCR primers used for the construction of His‐tagged GlgC, GlgC‐Long and GlgA1 expression plasmids.

No.	Label	Sequence
1	GlgA1.FOR	GCCTGGTGCCGCGCGGCAGCATGAAGATTTTATTTGTGGCGGCGGAAGTATCCC
2	GlgA1.REV	TGTCGACGGAGCTCGAATTCTTAGCGATAGGAAGCAGTTAACTCAGCGATTTTTTCCTCT
3	GlgC.FOR	TCATCATCATCACAGCAGCGGCCTGGTGCCGCGCGGCAGCGTGAAACGTGTCTTAGCGATTATCCTGGGCGG
4	GlgC.REV	CTCGAGTGCGGCCGCAAGCTTGTCGACGGAGCTCGAATTCCTAGATTACCGTGCCGTCGGCGATCGT
5	pET28a‐GlgA1.FOR	GCCACAAATAAAATCTTCATGCTGCCGCGCGGCACC
6	pET28a‐GlgA1.REV	TAACTGCTTCCTATCGCTAAGAATTCGAGCTCCGTCGACAAGCTTGC
7	pET28a‐GlgC.FOR	GCTGCCGCGCGGCACC
8	pET28a‐GlgC.REV	GAATTCGAGCTCCGTCGACAAGCTTGCG
9	slr1176NdeI (fw)	CATATGGTGTGTTGTTGGCAATCG
10	slr1176BamHI (rev)	GGATCCCTAGATTACCGTGCCGTC

To produce the GlgC‐long protein, the coding sequence of *slr1176* was amplified using primers 9 and 10, which contain appropriate extensions for the subsequent cloning (Table 3). The PCR product was cloned into pGEMT (Promega, Walldorf, Germany). DNA from sequence‐positive clones was extracted and the gene was cut out by restriction with *Nde*I and *Bam*HI. This fragment was then ligated with *Nde*I/*Bam*HI cut pET28a.

Production cultures of Rosetta‐gami cells with expression constructs were grown in batches of 2 L under standard conditions with 50 μg·mL^−1^ kanamycin for 4 h to OD_600_ = 0.6, then chilled at 4 °C for 10 min to bring down the culture temperature prior to induction. 1 mm isopropyl‐b‐d‐thiogalactoside (IPTG) was then added to the culture medium followed by incubation for 20 h at 18 °C with shaking at 125 rpm.

Cells were harvested by centrifugation at 6000 ***g*** for 13 min at 4 °C and flash frozen in liquid nitrogen for storage, or allowed to thaw at room temperature before resuspension and lysis in 50 mL of buffer containing 20 mm Tris/HCl pH 7.8, 50 mm NaCl, 5 mm MgCl_2_, 20 mm imidazole, DNase, RNase, lysozyme and one tablet of cOmplete™ Mini protein inhibitor cocktail (Roche, Mannheim, Germany). The His‐tagged proteins were purified using a desktop peristaltic pump with a 1 mL Ni‐NTA HisTrap columns (Cytiva, Freiburg, Germany) equilibrated with a buffer containing 20 mm Tris/HCl pH 7.8, 50 mm NaCl, 5 mm MgCl_2_, 20 mm imidazole. The column was washed with 5 and 3 column volumes (CVs) of buffer W1 (20 mm Tris/HCl pH 7.8, 500 mm NaCl, 40 mm imidazole) and W2 (20 mm Tris/HCl pH 7.8, 1000 mm NaCl, 80 mm imidazole), respectively before the His‐Tagged proteins were eluted with elution buffer (20 mm Tris/HCl pH 7.8, 500 mm NaCl, 300 mm imidazole). Positive fractions were selected using a Bradford test, pooled and subjected to a buffer exchange with dialysis buffer (20 mm Tris/HCl pH 7.8, 150 mm KCl, 1 mm EDTA, 50%_(v/v)_ glycerol) and a 3 kDa cut‐off regenerated cellulose tube. All purification steps were verified via SDS/PAGE and qualitatively prior to any large‐scale purification using a His‐SpinTrap kit (Cytiva) and the aforementioned buffers, following the manufacturer's instructions.

### Malachite green assay for GlgC activity

The activity of GlgC was assayed in the ADP‐Glc synthesis direction using a malachite green assay kit (Sigma‐Aldrich, MO, USA) and modification of a previously reported colorimetric method [7, 15]. Assays were performed in assay buffer containing 50 mm HEPES‐NaOH, pH 8.0, 12 mm MgCl_2_, and variable metabolite concentrations. The standard concentrations of the following metabolites (for a standard GlgC reaction) in a total reaction volume of 200 μL are, 2 mm 3‐PGA, 2 mm ATP, 2 mm Glc‐1P, 1 U·mL^−1^ PPiase (Thermo Fisher Scientific, Sindelfingen, Germany) and 5 μg·mL^−1^ of GlgC protein. All reactions were performed in 1.5 mL tubes at 30 °C, with shaking for 30 min. Reactions were started with the addition of substrate Glc‐1P or GlgC protein, depending on assay design. 8 μL of reaction mix was removed at specific timepoints for the malachite green assay, following the manufacturer‐supplied protocol.

### ADP/NADH coupled assay for GlgA1 activity

The activity of GlgA1 was evaluated using a modified spectrophotometric method from that presented by Wayllace *et al*. [19]. Assays were performed in assay buffer (50 mm HEPES‐NaOH, pH 8.0, 12 mm MgCl_2_) with 10 U·mL^−1^ Lactate Dehydrogenase (LDH), 5 U·mL^−1^ Pyruvate Kinase (PK), 1 mm Phosphoenolpyruvate (PEP), 0.4 mm NADH, 1 mg·mL^−1^ Rabbit Glycogen and 10 μg·mL^−1^ GlgA1 protein. The reaction was assayed in 96‐well clear bottom plates at 30 °C for 60 min. Absorbance was measured using Tecan Spark 10M (Tecan, Männedorf, Switzerland) every minute at 340 nm. Each reaction was started with the addition of 2 mm ADP‐Glc to a total assay volume of 200 μL.

### GlgC‐GlgA1 coupled glycogen synthesis assay

The entire glycogen synthesis reaction couples the synthesis of ADP‐Glc by GlgC with the GlgA1 reaction. Assays were performed in assay buffer with the components described above with 2 mm ATP, glycogen synthesis enzymes were added in a concentration ratio of 10 μg·mL^−1^ GlgA1 and 1 μg·mL^−1^ GlgC. Varying concentrations of test metabolites were according adjusted to reflect each assays design. The reaction was assayed and measured in 96‐well clear bottom plates as described above, beginning with the addition of Glc‐1P to a total assay volume of 200 μL.

### Mass photometry analysis

The procedure for mass photometry analysis was described in our previous work [32]. In brief, microscope coverslips (No. 1.5H, 24 × 5 mm, Marienfeld, Lauda‐Königsfeld, Germany) were prepared via immersion in isopropanol and ultrapure water. CultureWell gaskets (3 mm × 1 mm, Grace Bio‐Labs, Bend, OR, USA) were then applied to the coverslips. Prior to measurement, samples were diluted to 200 nm in assay buffer. Measurements used a TwoMP mass photometer (Refeyn, Oxford, UK) with AcquireMP v2022 R1 software, preceded by calibration. Focus was established by adding 5 μL buffer to a well, using an autofocus system based on total internal reflection. For acquisition, 5 μL of diluted protein was added, and 60‐s movies were recorded. Each sample was measured in triplicate. Images were processed and analysed using the DiscoverMP v2022 R1 software as described in Kofinova *et al*. [33].

### Figures, bioinformatics and analyses

All results were analysed and visualised using graphpad prism 10 (GraphPad, Boston, MA, USA). *In silico* design of primers and expression constructs were all performed with Snapgene (GSL Biotech, Boston, MA, USA). Figure 2A was created with Biorender (Biorender.com). GlgC structure prediction was performed with alphafold2 and visualised using chimerax [34, 35].

## Conflict of interest

The authors declare no conflict of interest.

## Author contributions

KL: designed and carried out experiments and wrote paper. SD: mass photometry experiments and drawing MH: construction of GlgC‐Long. KF: conceptualisation, supervision and paper writing. All authors contributed at varying stages in the editing and review of the paper.

### Peer review

The peer review history for this article is available at https://www.webofscience.com/api/gateway/wos/peer‐review/10.1111/febs.17348.

## Data Availability

Enzymatic data generated during this study have been deposited here (DOI: 10.15490/fairdomhub.1.study.1296.1) on the data and model management platform FAIRDOMHub [36].
